# Paciente masculino con cariotipo 46 XX negativo para el gen *SRY* y sin ambigüedad genital: reporte de un caso

**DOI:** 10.7705/biomedica.4687

**Published:** 2019-12-30

**Authors:** Andrea Casas-Vargas, Johanna Galvis, Jenny Blanco, Laura Rengifo, William Usaquén, Harvy Velasco

**Affiliations:** 1 Grupo de Genética de Poblaciones e Identificación, Instituto de Genética, Universidad Nacional de Colombia, Bogotá, D.C., Colombia Universidad Nacional de Colombia Grupo de Genética de Poblaciones e Identificación Instituto de Genética Universidad Nacional de Colombia BogotáD.C Colombia; 2 Grupo de Genética Clínica, Maestría en Genética Humana, Instituto de Genética, Universidad Nacional de Colombia, Bogotá, D.C., Colombia Universidad Nacional de Colombia Maestría en Genética Humana Instituto de Genética Universidad Nacional de Colombia BogotáD.C Colombia; 3 Grupo de Citogenética, Instituto de Genética, Universidad Nacional de Colombia, Bogotá, D.C., Colombia Universidad Nacional de Colombia Grupo de Citogenética Instituto de Genética Universidad Nacional de Colombia BogotáD.C Colombia

**Keywords:** trastornos testiculares del desarrollo sexual 46 XX, gen *SRY*, trastornos del desarrollo sexual, diferenciación sexual, secuencias repetidas en tándem, amelogenina., 46,XX testicular disorders of sex development, genes, *SRY*, disorders of sex development, sex differentiation, tandem repeat sequences, amelogenin.

## Abstract

En la mayoría de los casos, la diferenciación sexual masculina ocurre con la participación del gen SRY. Sin embargo, se pueden presentar otros genotipos excepcionales, como en el caso que se presenta en este reporte.

Se trata de un paciente adulto de sexo masculino atendido en el Servicio de Paternidades del Instituto de Genética de la Universidad Nacional de Colombia. Se le hicieron los análisis del gen de la amelogenina y de repeticiones cortas en tándem (*Short Tandem Repeat*, STR) específicas para el gen *SRY* con estuches comerciales de identificación humana, así como los de cariotipo convencional e hibridación *in situ* fluorescente del SRY, y el estudio de microdeleciones del cromosoma Y mediante reacción en cadena de la polimerasa (PCR). Se le hizo la evaluación clínica y se le brindó asesoramiento genético. El paciente no presentaba ambigüedad genital, su cariotipo era 46 XX, y el perfil molecular era negativo para el gen *SRY* y positivo para el *ZFY*. Se le diagnosticó un trastorno de diferenciación sexual 46 XX testicular no sindrómico, una rara condición genética. Solo el 20 % de los pacientes con este diagnóstico son negativos para *SRY* y exhiben perfiles moleculares diversos. La información disponible parece indicar que el *ZFY* está relacionado con la diferenciación sexual masculina, aún en ausencia del gen *SRY*.

## Abstract (en)

In most cases, male sexual differentiation occurs with *SRY* gene mediation. However, exceptional genotypes have been identified, as shown in this paper. This was a male adult patient seen at the *Servicio de Paternidades, Instituto de Genética*, *Universidad Nacional de Colombia*. The following procedures were carried out: Amelogenin gene and short tandem repeat analyses using human identification commercial kits, conventional karyotype, *SRY* fluorescent in situ hybridization, PCR analysis for Y chromosome microdeletions, clinical evaluation, and genetic counseling. We present an adult male with unambiguous genitalia, karyotype 46,XX, and an SRY negative and *ZFY* positive molecular profile. The diagnosis of nonsyndromic 46,XX testicular disorder of sex development (DSD) -a rare genetic condition- was established. Only 20 % of similarly diagnosed patients are SRY negative and exhibit diverse molecular profiles. Until now, available evidence seems to indicate that, even in the absence of *SRY*, the *ZFY* factor is involved in male sexual differentiation.

En el desarrollo sexual existen dos procesos principales: uno es la determinación sexual, es decir, lo que dirige el embrión indiferenciado hacia el desarrollo de la gónada bipotencial. El segundo es la diferenciación sexual, la cual ocurre por acción de los factores producidos por las gónadas ^1^. En los mamíferos -incluidos los humanos- se conoce ampliamente que el factor de la región de determinación sexual del cromosoma Y (*Sex-Determining Region Y*, *SRY*) cumple un papel clave en el desarrollo del testículo a partir de la gónada indiferenciada ^2^.

Por otro lado, la teoría clásica dicta que la diferenciación ovárica es una vía ‘por defecto’ que se ve inhibida en presencia del *SRY*. Sin embargo, en algunas contadas ocasiones, la diferenciación testicular puede ocurrir incluso en ausencia del cromosoma Y, lo cual da lugar a individuos 46 XX con fenotipo sexual masculino ^3^. Este tipo de síndromes genéticos es raro y se caracterizan por una discrepancia completa o parcial entre el sexo genético y el sexo fenotípico ^4^.

Aproximadamente, el 80 % de los pacientes con 46 XX y trastorno testicular del desarrollo sexual son positivos para el *SRY* y, usualmente, tienen un fenotipo masculino normal al nacer ^5^. El otro 20 % de hombres 46 XX son negativos para el *SRY* (*SRY*-) y muestran diferentes grados de masculinización, portan diferentes fenotipos y, a menudo, son estériles debido a la ausencia de las regiones *AZFa*, *AZFb* y *AZFc*^6^.

Asimismo, en el humano existen dos genes que permiten determinar el sexo cromosómico del individuo, uno es el *AMELX*, localizado en el cromosoma Xp22,1-22,3, el cual mide 2.872 pares de bases (pb), y el *AMELY*, ubicado en el cromosoma Yp11,2, de 3.272 pb. Estos dos genes se emplean usualmente en los análisis forenses, y en ellos se obtienen fragmentos de 106 pb y 112 pb, respectivamente ^7^.

Se presenta aquí el caso de un individuo con cariotipo 46 XX negativo para *SRY*, así como su discusión a la luz de la literatura.

Caso clínico

Un paciente de fenotipo masculino, de 40 años de edad, acudió al Grupo de Genética de Poblaciones e Identificación Humana del Instituto de Genética de la Universidad Nacional de Colombia, para hacerse una prueba de paternidad.

Con el fin de establecer la paternidad, se tomó una muestra sanguínea en un tubo con ácido etilendiaminotetraacético (EDTA), que luego se goteó sobre una tarjeta FTA Whatman™; el ADN se extrajo según el protocolo de la *Food and Drug Administration* (FDA) y se amplificó mediante reacción en cadena de la polimerasa (PCR) múltiple usando el estuche comercial Identifiler™ de Applied Biosystems, el cual contiene 15 marcadores genéticos autosómicos del tipo de repeticiones cortas en tándem (*Short Tandem Repeat*, *STR*) y un marcador para el gen de la amelogenina que permite determinar el sexo en la muestra analizada.

El siguiente paso fue la electroforesis capilar en el secuenciador ABIPRISM 310™ (LabX), en tanto que los resultados se analizaron con los programas GeneScan y TaqMan Genotyper Software, para obtener el electroferograma con el perfil genético del paciente, en el cual se observó la ausencia de amplificación correspondiente al marcador genético amelogenina del cromosoma Y (figura 1 ). El resultado se confirmó realizando todo el proceso nuevamente.

**Figura 1.**
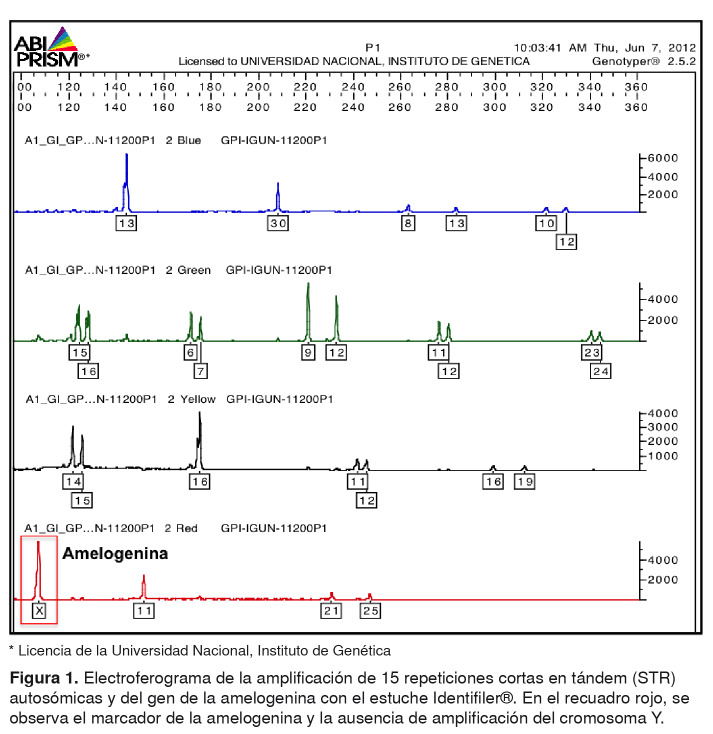
Electroferograma de la amplificación de 15 repeticiones cortas en tándem (STR) autosómicas y del gen de la amelogenina con el estuche Identifiler®. En el recuadro rojo, se observa el marcador de la amelogenina y la ausencia de amplificación del cromosoma Y

Asimismo, se amplificaron 16 microsatélites (STR) para el cromosoma Y empleando el estuche Yfiler™ (Applied Biosystems), pero no se obtuvo el perfil genético de dicho cromosoma (no se presentan los datos), lo que sugería una deleción del sitio de anillamiento de los iniciadores o la posible ausencia del cromosoma Y.

A continuación, se amplificó el gen *SRY* mediante PCR para un fragmento de 231 pb, empleando iniciadores específicos previamente publicados ^7^^,^^8^. Las secuencias de estos oligonucleótidos fueron las siguientes: SRY I: 5’-GGTCAAGCGACCCATGAAYGCNTT-3’ y SRY II: 5’-GGTCGATACTTATAGTTCGGGTAYTT-3’. En la amplificación se empleó la mezcla maestra HotStarTaq Master Mix™ (Qiagen) 2X, y 0,8 µM de cada iniciador.

El programa de PCR utilizado fue el siguiente: un ciclo de desnaturalización inicial a 95 °C durante 15 minutos, 30 ciclos de desnaturalización a 95 °C durante 30 segundos, un ciclo de anillamiento a 55 °C durante 30 segundos, uno de elongación a 72 °C durante 30 segundos y una elongación final a 72 °C durante 10 minutos.

El producto se visualizó mediante electroforesis en gel de agarosa al 1,5 % teñido con Syber Safe™ (Invitrogen). Se confirmó el tamaño molecular de la banda con el patrón de peso molecular HyperLadder II™ (Bioline). Al comparar los resultados de la amplificación con el control positivo, no se observó la amplificación de esta región en la muestra estudiada.

En la valoración clínica, el paciente refirió ser fruto de la undécima gestación de padres no consanguíneos (figura 2). El parto fue domiciliario, en zona rural, y el paciente tenía escaso conocimiento de los antecedentes de la madre, quien, al parecer, falleció por complicaciones asociadas con el último parto. Según su propio relato, tuvo un neurodesarrollo normal, cursó la primaria básica y no continuó sus estudios por motivos socioeconómicos.

**Figura 2.**
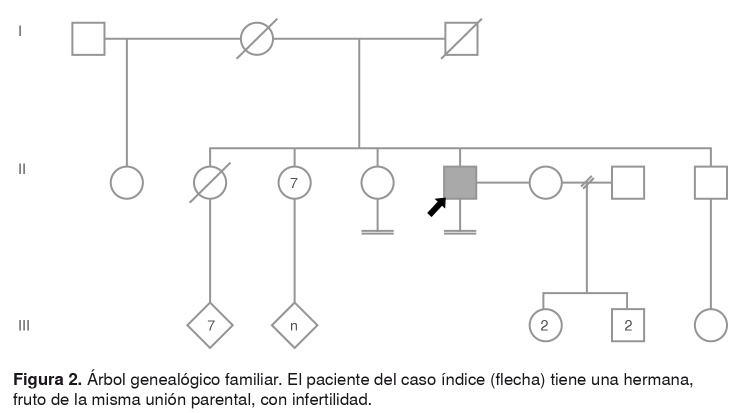
Árbol genealógico familiar. El paciente del caso índice (flecha) tiene una hermana, fruto de la misma unión parental, con infertilidad.

El paciente refirió haber desarrollado caracteres sexuales masculinos a los 13 años, aproximadamente, y negó haber presentado ginecomastia o hematuria. Refirió que sus parejas han sido todas mujeres y negó haber presentado síntomas de disfunción sexual. Desde hace 15 años tiene una pareja estable con la cual no ha concebido hijos. En cuanto a sus antecedentes familiares, refirió tener una hermana de 60 años con infertilidad, que no ha sido estudiada.

En el examen físico, se observó que el paciente presentaba el fenotipo masculino, con una talla de 1,56 m (puntuación estándar, *Z score*, de -2,7) y peso de 59,7 kg. Los signos vitales eran normales, no presentaba facies dismórfica, su vello facial era androgénico y no tenía ginecomastia. Sus genitales externos eran masculinos, con falo de 8 cm, testículos de 4 ml^3^ , descendidos en el escroto y sin masas, distribución del vello púbico androide y etapa V en la escala de Tanner. No se registraron hallazgos que sugirieran la presencia de alguna enfermedad (figura 3).

**Figura 3.**
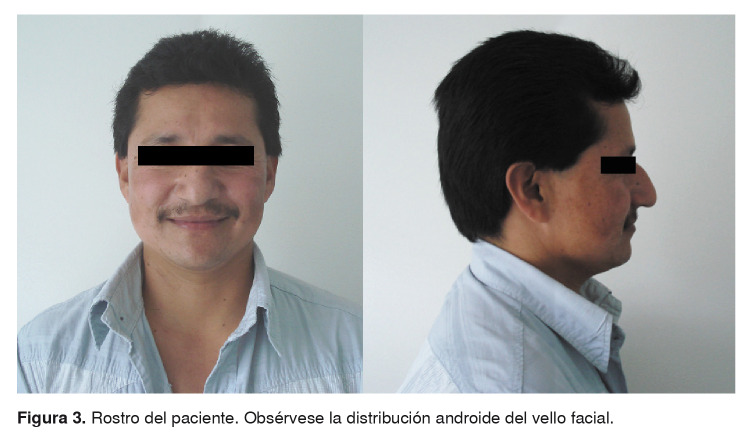
Rostro del paciente. Obsérvese la distribución androide del vello facial.

Se solicitaron los siguientes exámenes de apoyo clínico: una ecografía pélvica con reporte normal, en la cual se evidenció crecimiento prostático y ausencia de útero u otras estructuras mullerianas; la testosterona total fue de 6,10 ng/dL (valor de referencia en hombres hasta los 49 años: 2,4-10,8 ng/dL); el estradiol, de 32,46 pg/mL (valor de referencia en hombres: 13-54 pg/mL), y la 17-hidroxiprogesterona, de 1,81 ng/ml (valor de referencia en hombres 0,6-3,3 ng/ml). Los valores de los electrolitos (mEq/L) fueron: calcio, 9,8 (normal); cloro, 111 (normal); potasio, 5,3 y sodio, 147 (normales).

No se registraron signos clínicos ni historia de ambigüedad genital. Una nueva muestra del paciente fue remitida al Grupo de Citogenética y, a partir de ella, se determinó el cariotipo 46 XX (figura 4). Además, se hizo el análisis por hibridación fluorescente *in situ* (*Fluorescent In Situ Hybridization*, FISH) del gen *SRY*, el cual fue negativo [46 XX; ish (*DXZ*1x2, *SRY*-)] (figura 5).

**Figura 4.**
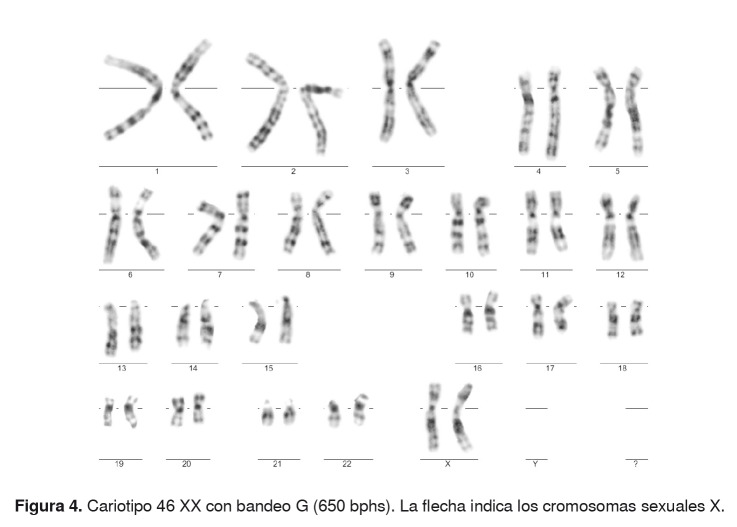
Cariotipo 46 XX con bandeo G (650 bphs). La flecha indica los cromosomas sexuales X

**Figura 5.**
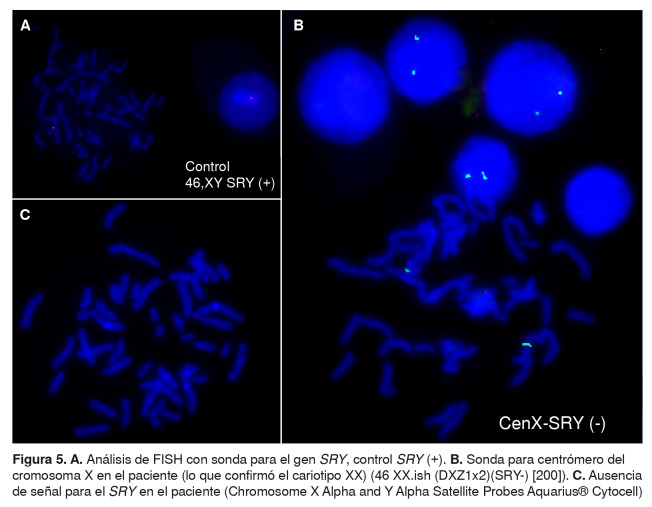
A. Análisis de FISH con sonda para el gen *SRY*, control *SRY* (+). B. Sonda para centrómero del cromosoma X en el paciente (lo que confirmó el cariotipo XX) (46 XX.ish (DXZ1x2)(SRY-) [200]). C. Ausencia de señal para el *SRY* en el paciente (Chromosome X Alpha and Y Alpha Satellite Probes Aquarius® Cytocell)

Por último, se estudiaron las microdeleciones del cromosoma Y mediante PCR y, siguiendo las directrices de la *European Academy of Andrology* (EAA) y la *European Molecular Quality Network* (EMQN) ^9^, se amplificaron cinco regiones específicas y se obtuvieron los siguientes resultados: *SRY*-, *ZFY*+ (homólogo de *ZFX*), *AZFa*-, *AZFb*- y *AZFc*-, lo que evidenció la ausencia completa del cromosoma Y en el paciente.

En una entrevista posterior, se le entregó al paciente el informe clínico y se le explicó su diagnóstico: trastorno de diferenciación sexual 46 XX testicular no sindrómico (anteriormente conocido como síndrome de reversión sexual 46 XX). El paciente firmó el consentimiento informado para la publicación de su caso y autorizó por escrito la toma de fotografías de su rostro.

El resultado de la prueba de paternidad fue de ‘exclusión’. El paciente refirió haberse practicado con anterioridad este mismo examen en otra situación de presunta paternidad, el cual arrojó el mismo resultado. Se le explicó que su diagnóstico molecular de ausencia del cromosoma Y generalmente implica infertilidad. El paciente no asistió a la cita de control para recibir el resultado del espermograma y cambió sus datos de contacto sin reportarlo a la institución, por lo cual no fue posible continuar con el seguimiento.

## Discusión

El trastorno de diferenciación sexual 46 XX testicular no sindrómico es una condición infrecuente en la población, con una incidencia de 1 en 20.000 nacidos vivos ^5^. En la base de datos *Online Mendelian Inheritance in Man* (OMIM) se encuentra esta condición bajo el nombre de reversión sexual 46 XX (OMIM 400045).

Se cree que en los pacientes positivos para el *SRY* (*SRY+*) el cruce entre las regiones pseudoautosómicas de los cromosomas sexuales, el cual ocurre durante la meiosis paterna, puede causar una translocación resultante en este tipo de trastorno de diferenciación sexual ^10^. En los pacientes negativos para el *SRY* (*SRY*-), las causas son más complejas. Algunos autores han sugerido que hay diversos genes que contribuyen a la determinación del sexo, localizados en autosomas que inician la ‘masculinidad’ ^11^^,^^12^, lo cual sigue siendo materia de investigación.

En este caso, se hicieron exámenes de laboratorio para descartar otros posibles diagnósticos y se encontraron niveles normales de 17-hidroxiprogesterona, testosterona y estradiol. La ecografía pélvica no evidenció estructuras mullerianas ni remanentes de estas. Además, los electrolitos fueron normales. Por todo ello, se excluyó el diagnóstico de hiperplasia suprarrenal congénita, la causa más frecuente de virilización en individuos XX ^5^. Otro diagnóstico diferencial es la translocación del gen *SRY*^10^, lo cual se excluyó mediante los análisis de STR y de FISH. Por lo tanto, se consideró que el paciente hace parte del 20 % de individuos masculinos XX negativos para el *SRY*, es decir, que no poseen el gen *SRY* o carecen, por lo menos de forma sustancial, del material genético del cromosoma Y ^5^^,^^11^^,^^12^.

En estudios recientes se ha confirmado que, incluso en ausencia del *SRY*, puede ocurrir una diferenciación sexual masculina completa por sobreexpresión de genes como el *Sox9*, por reordenamientos del *Sox3*, o debido a mutaciones con pérdida de función en los genes *Wnt4* y *Rspo1*^13^^,^^14^. En cuanto al fenómeno contrario, es decir, la reversión sexual masculina a femenina, en el ratón y en el humano existe un *locus* de reversión sexual sensible a la dosis ligado a X, el cual funciona como un represor de la vía masculina: el *DAX1*^12^^,^^14^. Sin embargo, en el modelo de ratón, Meeks, *et al*., confirmaron que las mutaciones en el gen *Dax1* conducirían, paradójicamente, a una reversión del sexo femenino a masculino ^14^, pero en humanos no se ha reportado aún este fenómeno.

En diversos artículos se han descrito tres grupos diferentes del trastorno de diferenciación sexual 46,XX testicular *SRY*- según las características fenotípicas. El paciente del presente caso se ajustaba al grupo clásico de hombres XX fenotípicamente normales (es decir, sin ambigüedad genital ni hermafroditismo); además, su talla era baja y presentaba hipogonadismo discreto, lo cual concuerda con reportes de casos similares en la literatura mundial ^15^^-^^18^.

En todos los casos reportados se registró invariablemente la esterilidad, pero los perfiles moleculares han sido variables ^16^. Rajender, *et al*., reportaron un caso de reversión sexual 46 XX/*SRY*-, en el cual la causa molecular no pudo esclarecerse: la secuenciación y el análisis de la variación del número de copias del *SOX9* fueron normales, así como la secuenciación de la proteína DAX1, y con la PCR se excluyó la presencia de *ZFY*, *AZFb* , *AZFc* o cualquier otro material proveniente del cromosoma Y ^15^. En otro reporte de caso, dos hombres con azoospermia, miembros de una misma familia, presentaron un perfil 46 XX/*SRY*- , así como una triplicación de una región de 500 kb corriente arriba del gen *SOX9*. Los investigadores plantearon la hipótesis de que elementos reguladores allí presentes pudieran resultar afectados por dicha amplificación, produciendo de esta manera el fenotipo ^17^. Por el contrario, otros investigadores presentaron una ganancia similar en otro individuo y la clasificaron como un polimorfismo no relacionado con la clínica ^18^.

En el paciente de nuestro caso, se detectó mediante PCR la presencia del gen *ZFY*, el cual se ubica normalmente en el brazo corto del cromosoma Y (Yp11.2) y es homólogo del gen *ZFX*^19^. También mediante PCR, Palmer, et al., demostraron similitudes entre los genes *ZFY* y *ZFX*, los cuales se expresaron en tejidos fetales y adultos, en tanto que el *ZFX* se expresó en el cromosoma X inactivo presente en híbridos de ratón y humano ^20^. El comportamiento evolutivo de estos dos genes ha permitido estudiar su papel en la diferenciación sexual, concretamente en la aparición del cromosoma Y en los vertebrados ^21^.

Los casos de *ZFY*+ detectados mediante PCR podrían responder a una translocación del *ZFY* en uno de los autosomas, o en uno de los cromosomas X ^22^, pero es más probable que se deban a un evento de amplificación no específica, debido a que los cebadores empleados de manera consensuada pueden amplificar tanto el *ZFY* como el *ZFX*^9^.

En un estudio de Tian, *et al*., de 14 pacientes con trastorno de diferenciación sexual y cariotipo 46,XX, se identificó un perfil *SRY*-/*ZFX*+ en tres de ellos, en tanto que siete resultaron *SRY*+/*ZFY*+. Todos estos individuos presentaban grados variables de ambigüedad genital ^16^ y ninguno de ellos presentaba un perfil 46 XX *SRY*-/*ZFY*+ , tal como sucedía en el paciente colombiano del presente reporte.

A pesar de que no se pudo contar con nuevas muestras para precisar mejor el diagnóstico del paciente, es pertinente mencionar que el empleo de tecnologías de nueva generación, como la *Next Generation Sequencing* (NGS), los paneles para la detección de trastornos del desarrollo sexual, o la secuenciación del exoma facilitarían llegar a una conclusión etiológica.

## Conclusión

La disponibilidad de pruebas de diagnóstico molecular ha permitido investigar las posibles causas del trastorno de diferenciación sexual 46 XX testicular detectado en el paciente aquí descrito. Su perfil molecular es *SRY* negativo/*ZFY* positivo y, pese a los pocos reportes clínicos de las mismas características, los datos disponibles parecen indicar que esta amplificación no fue específica debido a limitaciones propias de la técnica. El empleo de la NGS en estos casos podría aportar más elementos para confirmar el diagnóstico genético, y facilitaría el asesoramiento y el pronóstico reproductivo.
